# Effect of Vif in doxorubicin treated breast cancer cells

**DOI:** 10.1080/07853890.2021.1895582

**Published:** 2021-09-28

**Authors:** Susana Bandarra, Pedro Cipriano, João Gonçalves, Ana Clara Ribeiro, Isabel Barahona

**Affiliations:** a Laboratório de Biologia Molecular, Centro de investigação interdisciplinar Egas Moniz (CiiEM), Instituto Universitário Egas Moniz, Caparica, Portugal; b Faculdade de Ciências e Tecnologia, Universidade Nova de Lisboa (FCT-UNL), Caparica, Portugal; c Research Institute for Medicines (iMed.ULisboa), Faculty of Pharmacy, University of Lisbon, Lisbon, Portugal

## Abstract

**Introduction:**

Several studies linked DNA cytosine deaminase APOBEC3 to mutational process driving carcinogenesis [1]. However, APOBEC3 expression varies in breast cancer cells [2] and their role in breast cancer treatment remains elusive. The HIV-1 and HIV-2 Vif proteins are APOBEC3 specific inhibitors that recruit the host E3 ubiquitin ligase complex, inducing APOBEC3 ubiquitination and degradation in proteasomes [1]. In this work, our aim is to inhibit APOBEC3 using Vif and determine the sensibility of breast cancer cells to a non-hormonal treatment with doxorubicin.

**Materials and methods:**

Triple negative breast cancer cell line HCC1806 was transduced with lentiviruses containing Vif-1 and Vif-2 genes in fusion with ZsGreen reporter gene producing two different cell lines named as VIF1 and VIF2 cells. Before cells treatment with doxorubicin, the expression of the fluorescent marker and Vif was confirmed by Fluorescent Activated Cell Sorting (FACS-AriaIII) and PCR, respectively. Characterisation of doxorubicin dose-and time-responsive cell viability was performed using MTT assay.

**Results:**

High-titers of Vif-delivering lentiviruses were produced and used to transduce efficiently the HCC1806 cells. More than 99% of sorting population expressed ZsGreen indicating that Vif-1 and Vif-2 genes were integrated in genomic DNA and expressed in VIF1 and VIF2 cell lines. After treatment with doxorubicin for 24 h, all cell lines showed significant decrease of viability when compared with untreated cells, proportional to the concentration of doxorubicin (Figure 1). Comparison between cell viability of HCC1806 (parental) and VIF1 shows no difference in contrast with the behaviour of VIF2 cells that after doxorubicin treatment showed a significant increase in viability. **Discussion and conclusions:** The increased viability of doxorubicin treated VIF2 cells correspond to the development of cells resistance to doxorubicin. This resistance of VIF2 cells is probably related to the APOBEC3 inhibition by Vif 2 protein. Our results raise concerns about general use of doxorubicin as breast cancer treatment, especially when APOBEC3 expression is low.

**Figure 1.**
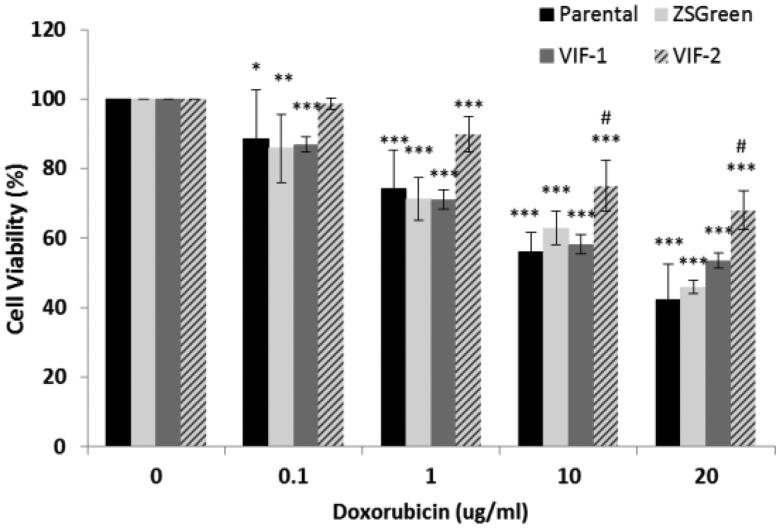
Viability of cells treated with doxorubicin for 24 h. Values are presented has mean ± SEM of three independent MTT assays. *p-value <.05 in relation to untreated cells from the same cell line. #*p*-value <.05 in relation to HCC1806 parental cell line.
